# Neuropsychological and psychological dysfunctions associated with coronavirus disease 2019: a case report

**DOI:** 10.1186/s13256-021-03104-w

**Published:** 2021-10-18

**Authors:** Maria Damianova

**Affiliations:** 1 Neurosurgery Clinic, University Multiprofile Hospital for Active Treatment “Saint Ivan Rilski”, 15 Akademik Ivan Geshov Blvd., Sofia, Bulgaria; 2 School of Human and Community Development (Department of Psychology), University of the Witwatersrand, Johannesburg, South Africa; 3 School of Clinical Medicine, University of the Witwatersrand, Johannesburg, South Africa

**Keywords:** COVID-19, Neuropsychological and psychological dysfunctions, Cognitive impairment, Executive dysregulation, Mood disturbances

## Abstract

**Background:**

Patient’s account of personal experiences of having lived through coronavirus disease 2019 is important for understanding the magnitude of the debilitating impact of the infection. There is increasing recognition that the infection impedes multiple functional domains, but to date the evidence remains scarce. Moreover, to the author’s knowledge, there are no documented cases reporting on research data derived from self-reflective first-person experience.

**Case presentation:**

The patient was a 59-year-old female psychologist of White self-ascribed ethnicity who had coronavirus disease 2019. She had no history of medical, neurological, or psychiatric conditions and works in a neurosurgery clinic at a large hospital as a psychologist, specializing in neuropsychology. Using the introspective method, she captured the occurrence of neuropsychological and psychological dysfunctions she experienced in the acute stage of the illness, which took place in December 2020 and lasted for 17 days. Treatment of coronavirus disease 2019 was conducted in the home environment under medical supervision and followed a standardized protocol adopted at the time in the country.

**Conclusions:**

The data derived from the first-person experience indicated that among the most salient cognitive functions impacted by the disease were: executive control, working memory, attention, concentration, and processing speed.

Furthermore, emotional instability; mood swings; racing, repetitive, or intrusive thoughts; uncontrolled associations; dizziness; fatigue; disbalance; and sleep disturbances featured consistently throughout the illness. The overall profile of these dysfunctions suggests disruption in the overall operation of the brain and particularly in the functioning of the frontal lobes. Although less tangible than the physical symptoms, the neuropsychological and psychological dysfunctions associated with coronavirus disease 2019 form a distinct cluster that has a highly debilitating impact on a person’s well-being.

## Background

The impact of coronavirus disease 2019 (COVID-19) on the functioning of the brain is progressively being underscored as societies accumulate both scientific and experiential knowledge of living through the illness. Lee *et al.* [1] reported findings of multifocal microvascular brain injuries in a postmortem study of brain tissue from patients with COVID-19. In a sample of 125 patients, Varatharaj *et al.* [2] reported that a large proportion (62%) of patients presented with a cerebrovascular event and 31% presented with altered mental status, of which 23% had unspecified encephalopathy and 18% had encephalitis. In their study, as many as 59% of the patients who manifested altered mental status fulfilled the diagnostic criteria for psychiatric diagnoses. It has been proposed that anxiety is at the core of the predisposing, precipitating, and perpetuating factors underlying various neuropsychiatric abnormalities (such as agitation, disorganization, paranoid ideation, and auditory hallucinations) detected in patients with COVID-19 [3]. Metabolic disturbances and white matter damage in the brain due to hypoxia were detected in a recent study by Rapalino *et al.* [4]. A variety of studies (see [5] for a detailed review) have shown that severe acute respiratory syndrome coronavirus 2 (SARS-CoV-2) infection may present with various neurological and central nervous system (CNS) symptom manifestations. Among these are headache and confusion, and even seizures or encephalitis. Ardila and Lahiri [6] specifically anticipated the occurrence of executive dysfunction as a part of the neurological impairments due to the infection, in both the acute phase and the long run. Lima *et al.* [5] proposed that the virus may invade the CNS through various pathways within dual (hematogenous and neuronal) routes. In this transmission, the olfactory pathway in particular is thought to play a significant role. The findings of Zhou *et al.* [7] highlighted the parallel organization of the olfactory system and indicated that the anterior olfactory nucleus has the strongest unique connectivity with the orbitofrontal cortex. Other smaller connectivity clusters were found to reside in the left inferior temporal gyrus, bilateral anterior temporal gyri, the bilateral anterior insula and the mammillary bodies, and retromammillary commissure. Furthermore, their work revealed that, compared with other sensory systems, the olfactory networks reach a broader set of cortical targets (largely situated in the frontal and temporal piriform cortices) at an earlier stage of information processing. This functional specificity along with the strong frontotemporal anatomical brain connectivity of the olfactory system is likely to underlie the key role of the latter for the invasion of the viral infection into the CNS. Contributing factors to the wide scope of neurofunctional abnormalities brought about by the virus could be its neuroinvasive, neurotropic, and neurovirulent nature [5] and the multiple pathways through which it attacks the CNS. In their recent study, conducted with COVID-19 patients during acute inpatient rehabilitation stage prior to discharge, Jaywant *et al.* [8] found that attention and executive functions were most affected. This finding lends support to the view put forward by Snyder *et al.* [9] that executive control impairments are transdiagnostic and inherent to a broad spectrum of clinical conditions.

The evaluation of neuropsychological and psychological functions, while the person is in the acute stage the illness, presents a methodological challenge. Firstly, the primary focus in the ongoing treatment is preservation of life and ensuring the patient’s survival. Secondly, the patient’s overall health status is likely to not permit them to participate in the assessment process. Thirdly, the administration of the neuropsychological tests typically requires face-to-face interaction, which is not possible given the risk of contamination. Consequently, systematic data collection using psychometric tests is not feasible. Introspective inquiry capturing the first-person experience of trained psychologists and neuropsychologists who had COVID-19 is considered methodologically relevant and appropriate, particularly in view of the unfeasibility of applying psychometric methods for assessing the impairments emerging as the infectious process evolves.

The aim of the present investigation was to document the first-person account of the range of neuropsychological and psychological symptoms experienced by the author while undergoing COVID-19. Her reflections on the presenting symptoms and the formulation on their hypothesized generative mechanisms are informed by her extensive professional experience in conducting neuropsychological and psychological assessments and providing psychotherapeutic support to patients with various brain dysfunctions, including tumors, traumatic brain injuries (TBI), degenerative disorders such as Parkinson’s disease (PD), developmental disorders, epilepsy, functional neurological disorder (FND), and psychogenetic non-epileptic seizures (PNES).

## Case presentation

### Methodological considerations regarding the method of introspection and first-person experience

The method of introspection has a long history in the development of psychological science, dating back to the work of Wilhelm Wundt and William James. The method involves “the looking into our own minds and reporting what we there discover” [10], p. 185). Historically, introspection has been valued as it allows for gaining direct access to a person’s own mental states [11]. Despite its limitations, introspection remains a useful and ubiquitous approach to understanding a person’s inner reflections and intimate experiences. This claim is supported by the in-depth investigation by Rigato *et al.*, [11] of the utilization and scientific merits of the first-person experience method. Their extensive review of methodological approaches implemented in cognitive science led to the conclusion that over the years up to the present date this method has remained central to the study of the mind and is fundamental to cognitive science and cognitive neuroscience. In the same vein, Xue and Desmet [12] have put forward a strong argument highlighting the unique contribution of researcher self-introspection to advancing knowledge production. They emphasized that the first-person perspective is uniquely positioned to provide access to subjective experiences in naturalistic settings at a level of “readiness, vividness, richness and depth” (p. 42) that is not readily attainable by other methods of investigation and/or third-person perspective. Among the distinct strengths of the first-person perspective is not only data richness, readiness, and accessibility but depth of analysis and reflexivity as well. For these reasons, self-reflective accounts are particularly powerful and valuable for unpacking phenomena that are primarily of first-person nature and can thus be directly accessible only to a first-person mode of research [13], as is the present case. On these grounds, the first-person research mode, techniques, and procedures are deemed well befitting the inherent contextual specificities of this inquiry, namely: (a) the contaminating nature of the infection necessitating the patient’s isolation in the course of the illness, (b) scarcity of clinical knowledge of the infectious process and limited medical care expertise at the time, (c) newly standardized and continuously evolving treatment practices for COVID-19, (d) the patient’s professional background (qualified and experienced psychologist, neuropsychologist, and researcher).

## Patient information

The patient was a 59-year-old female, with no history of medical, neurological, or psychiatric conditions and with no predisposing, premorbid, or comorbid conditions of note. She is married and has a grown-up son. She works in a neurosurgery clinic at a large hospital as a psychologist and neuropsychologist. She is socially and professionally active, with vast experience in clinical and psychotherapeutic work, academia, and research.

### Diagnostic procedure and treatment

Daily personal experiences of the neuropsychological and psychological symptoms were documented during a 17-day period, starting from the initial occurrence of physical symptoms (temperature, general indisposition, and fatigue) on 18 December 2020 through to the confirmatory diagnosis via polymerase chain reaction (PCR) test on day 4 after the initial symptoms, and the subsequent negative PCR test outcome on day 15 post-diagnosis. Treatment of COVID-19 was conducted in isolation in the home environment under medical supervision and as per the standardized protocol in the country, which included daily intake of vitamins B, C, and D, a 10-day course of antibiotics, and a 7-day course of dexamethasone, as well as melatonin and aspirin at night. As the patient was isolated, external observations were not feasible and the clinical data reported below could only be generated from first-person self-reflections and self-observations. However, throughout the period of illness, the patient communicated verbally to the treating medical doctor daily telephonic reports on her self-reported symptomatology. These self-evaluative reports informed relevant and timeous patient-centered clinical interventions. Subsequent to the negative PCR test outcome, the patient continued experiencing the symptoms described below with fluctuating intensity and frequency for a period of approximately 3 months postmorbidly.

## Results

### Physical symptoms

The physical symptoms included heightened temperature ranging from 37.0 °C to 37.7 °C over 5 days prior to commencing with antibiotics treatment; headaches, joints, and muscle pain; loss of sense of smell for 3 days (starting on the first day after the initial symptoms); altered taste (unpleasant taste of metal and bitterness lasting throughout the illness); occasional daily periods of shortness of breath; tightness in chest.

### Neuropsychological and psychological symptoms

The range of neuropsychological and psychological impediments experienced by the patient pertains to the following domains:

#### Working memory and mental/double mental tracking

Mental operations and juggling with multiple pieces of information (both verbal and visuospatial) were disordered, and there was a frequent loss of train of thought.

#### Executive control

Most of the components of executive control were affected. Planning was poor, utilization of feedback was ineffective, structuring and organizing of daily activities were disarranged, and there were difficulties with initiating and/or stopping activities. Thought processes lacked both flexibility and stability, which was manifested by either losing logical and systematic order of thought processes or difficulties making mental shifts. The overall experience was that the interfunctional and intrafunctional organization and integration of cognitive, behavioral, and emotional systems was deranged and that the overall sense of self-control was shattered.

#### Processing speed

The pace of execution of activities was unstable and highly variable (either too accelerated or too slow).

#### Attention and concentration

There was a notable worsening of divided, focused, and sustained attention. Maintaining focus on the activity at hand or registering incoming information was defective. Experiences like “floating” and "lightheadedness" happened often, with variable duration.

#### Emotional regulation and self-control

Disinhibition, verbal outbursts of anger, and sense of helplessness and fear were at times overwhelming. As highlighted in the section “Mode of symptom occurrence,” the course of the illness was characterized by unpredictability of appearance of the symptoms and their erratic daily frequency and duration. “Good days/times” and “bad days/times” were random. The peaks and dips in symptom occurrence and intensity did not have a coherent pattern and could not be anticipated. In turn, this symptom fluctuation and variability further exacerbated the sense of shattered self-control.

#### Thought processes

Racing, intrusive, or repetitive random thoughts and uncontrolled associations as well as vivid images of movies and past life events were sudden, recurrent, and invasive.

#### Language

Isolated instances of semantic paraphrases and word retrieval difficulties for common words and for names in particular were manifested in spontaneous speech.

#### Mood

Mood was unstable, and mood swings ranging from apathy and inertia to elevated agitation and uncalled-for hyperactivity featured frequently. Anxiety was overwhelming and underlined the entire experience of the illness. Anxiety levels fluctuated and seemed related to the variability of experiencing improvement or deterioration in the overall mental and physical condition. Fear of not knowing what will happen next and whether one can overcome the illness was pervasive. The extreme, pendulum-like variations in the sense of wellness inflicted back-and-forth swings between hopefulness for recovery and despair about a potentially fatal outcome.

#### Sleep

Sleep was disrupted by periodic awakenings and by an invasion of random thoughts and visual images.

#### Fatigue, dizziness, and disbalance

At times, even simple physical activities like getting up and walking were difficult and required a significant effort. Episodes of dizziness and disbalance emerged randomly and with variable duration throughout the illness, thus enhancing the sense of physical and mental instability.

#### Mode of symptom occurrence

The occurrence and intensity of the symptoms described above fluctuated markedly over the 17-day period and then gradually subsided in the course of recovery, which lasted for approximately 3 months. Overall, the key features defining the mode of occurrence of the neuropsychological and psychological dysfunctions included: (a) random, rapid, sudden, and unpredictable invasion of symptoms; (b) extreme variations of symptom duration, daily frequency, and time of appearance; (c) variable and diverse combinations and co-occurrences of some or all symptoms within the same day. Thus, the daily incidence and the progression of symptoms over the 17-day duration of acute illness did not form any particular systematic pattern.

## Discussion

This investigation was carried out with the aim of documenting the neuropsychological and psychological impairments experienced by a trained psychologist in the course of her undergoing COVID-19. Using the introspective method encompassing self-analysis, self-observations, self-evaluations, and self-reflections on the first-person experience allows for formulating an in-depth account of the impairments of cognitive, behavioral, and emotional functions evolving within the progression of the infectious process. To the author’s knowledge, the current case presentation is the only first-person research account of neuropsychological and psychological dysfunctions associated with COVID-19, as experienced by a trained psychologist and neuropsychologist. The present study demonstrates the value of first-person methodological approach for accessing phenomena that are not readily attainable by any other methods of investigation or third-person perspective.

Compatible with the results documented by Jaywant *et al.* [8], the present findings indicate that the cluster of dysfunctions manifested in the course of COVID-19 infection pertain to core existential domains: working memory, attention, concentration, executive control, emotional regulation, and mood. Such a cluster would be typically implicated in and would point to vulnerability of and/or distortion in the operation of the frontal lobes. Taking into account the parallel organization of the olfactory system, its strongest unique connectivity with orbitofrontal cortex [7], the multiple routes for the invasion of the virus into the CNS, and the significant role of the olfactory system in this regard [5], it is likely that the viral infection brings about an impediment in the overall functional organization of the brain. The frontal lobes systems provide the major contributions to the integrity of the interfunctional and the intrafunctional organization of the brain. The profile of cognitive, emotional, and behavioral dysfunctions identified in this study is compatible with the proposition that the frontal lobes may be particularly vulnerable to the viral invasion. The hypothesized mechanisms underlying the overall neuropsychological and psychological instability brought about by COVID-19 appear to be related to the fragmentation of executive functioning, including the deconstruction of a person’s self-regulation capacities at both mental and physical levels. Considering that the executive control impairments have a wide spectrum of implications for psychopathology and cognitive dysfunctions [9], it is reasonable to contemplate that the manifested erratic mode of symptom occurrence and presentation depicts too an overarching distortion of executive functioning.

## Conclusions

The present study offers an explorative first-person analysis of subjective experiences of dysfunctions in cognition, emotionality, and behavior arising due to COVID-19. The methodological limitations pertain to the subjective essence of the data and the inherent proneness to bias [14]. Nevertheless, the findings can be considered a useful point of departure for generation of novel hypotheses [14], and for systematically and continuously collecting and integrating further neuropsychological, neurophysiological, and neuroanatomical data highlighting the multifarious debilitating impact of the disease. Given that the longer-term sequelae did not fall within the scope of this study, future research endeavors should aim at filling in this knowledge gap as well as extend the scientific inquiries using prospective designs and third-person methodologies. Though generated from an individual case, the insights, experiential knowledge, and in-depth reflections presented in this case report reveal a profile of COVID-19-related cognitive, emotional, and behavioral impairments for which very little is known so far.

The identification of the impairments in executive control, working memory, attention and concentration, processing speed, emotional regulation, and mood may also serve as a foundation for providing psychological counseling and advice to patients in both the acute and recovery stages of the illness. Guiding patients and managing their expectations throughout the emergence, expansion, and intensification of the presenting neuropsychological and psychological symptoms is likely to enhance patients’ resilience and coping mechanisms and minimize the debilitating impact of the infection.

## Data Availability

Data are first-person experiential reflections and self-observations.
